# PIP_2_ regulation of KCNQ channels: biophysical and molecular mechanisms for lipid modulation of voltage-dependent gating

**DOI:** 10.3389/fphys.2014.00195

**Published:** 2014-05-27

**Authors:** Mark A. Zaydman, Jianmin Cui

**Affiliations:** Department of Biomedical Engineering, Center for the Investigation of Membrane Excitability Diseases, Cardiac Bioelectricity and Arrhythmia Center, Washington University in St. LouisSt. Louis, MO, USA

**Keywords:** PIP_2_, voltage-gating, lipid modulations, ion channel, KCNQ

## Abstract

Voltage-gated potassium (Kv) channels contain voltage-sensing (VSD) and pore-gate (PGD) structural domains. During voltage-dependent gating, conformational changes in the two domains are coupled giving rise to voltage-dependent opening of the channel. In addition to membrane voltage, KCNQ (Kv7) channel opening requires the membrane lipid phosphatidylinositol 4,5-bisphosphate (PIP_2_). Recent studies suggest that PIP_2_ serves as a cofactor to mediate VSD-PGD coupling in KCNQ1 channels. In this review, we put these findings in the context of the current understanding of voltage-dependent gating, lipid modulation of Kv channel activation, and PIP_2_-regulation of KCNQ channels. We suggest that lipid-mediated coupling of functional domains is a common mechanism among KCNQ channels that may be applicable to other Kv channels and membrane proteins.

## Introduction

Lipids define the physical and chemical environment of voltage-gated ion channels, yet most of the literature in the ion channel field considers only the channel protein and its proteinacious interacting partners. This protein-centric view is a natural consequence of our powerful genetic tools for manipulating the primary sequence of ion channels and heterologously expressing mutants for functional study. In comparison, we have a limited set of tools for manipulating the lipid environment of ion channels in heterologous or native cells. Despite this limitation, the importance of non-specific (bulk) and specific (cofactor) lipid interactions to ion channel function is a subject of continuing interest. Phosphatidylinositol 4,5-bisphosphate (PIP_2_) is an anionic lipid found in the inner leaflet of the surface membrane where it makes up a small fraction (<1%) of the total pool of phospholipids (McLaughlin et al., 2002; Rusten and Stenmark, 2006). PIP_2_ is known to directly bind to and regulate a diverse set of ion channels (Hilgemann and Ball, 1996; Hilgemann et al., 2001; Suh and Hille, 2005, 2008). PIP_2_ regulation is an excellent model for studying the cofactor-lipid regulation of ion channels, as we have increasingly more tools to manipulate the abundance of PIP_2_ in heterologous cells (Suh et al., 2006; Suh and Hille, 2007a; Okamura et al., 2009). Additionally, changes in the abundance of PIP_2_ that are sufficient to alter channel activity are not likely to change the bulk membrane properties.

Voltage-gated ion channels are transmembrane proteins that sense membrane voltage and respond by opening or closing an ion conductive pathway across the membrane. The ionic currents from voltage-gated ion channels generate action potentials in excitable tissues; thereby, they control muscle contraction, neuronal signaling, immune activation, and neurohormonal secretion. Variations in the abundance of membrane PIP_2_ modulate the function of several families of voltage-gated ion channels, including K^+^ channels (Kv) (Bian et al., 2001; Loussouarn et al., 2003; Zhang et al., 2003; Oliver et al., 2004; Abderemane-Ali et al., 2012; Rodriguez-Menchaca et al., 2012), Ca^2+^ channels (Cav) (Wu et al., 2002; Gamper et al., 2004; Suh et al., 2010), hyperpolarization and cyclic nucleotide activated channels (HCN) (Pian et al., 2006; Zolles et al., 2006), and voltage and Ca^2+^-activated K^+^ channels (BK) (Vaithianathan et al., 2008). It should be noted that the physiological relevance of the PIP_2_-sensitivities of some of these channels remains controversial (Hilgemann, 2012; Kruse et al., 2012). Among these voltage- and PIP_2_-sensitive channels, the KCNQ (herein we use KCNQ to refer gene and protein product—i.e., Kv7) family is unique in that its members absolutely require PIP_2_ in order to conduct current (as discussed below) making them a relatively straight-forward model to study PIP_2_ regulation of voltage-dependent gating. The KCNQ family includes five voltage-gated potassium-selective channels (KCNQ1-5) that are known to play important roles in regulating cardiac action potential duration (Barhanin et al., 1996; Sanguinetti et al., 1996; Wang et al., 1996), modulating neuroexcitability (Wang et al., 1998), and maintaining endolymph K^+^ homeostasis in the inner ear (Neyroud et al., 1997). The physiological importance of PIP_2_ regulation of KCNQ channels is well established in neurons where silencing of KCNQ channel activity by PIP_2_ hydrolysis downstream of G-protein Gq signaling increases neuroexcitability (Delmas and Brown, 2005; Brown et al., 2007). The role of PIP_2_ modulation of native cardiac KCNQ1 channels is less well established. However, in the heart, α1 adrenergic receptors (α1 AR) activate Gq signaling pathways (Jensen et al., 2011) which has been shown in heterologous expression systems to modulate I_Ks_ channels (formed by KCNQ1 and the auxiliary subunit KCNE1) in through a combination of PIP_2_ hydrolysis and PKC phosphorylation (Matavel and Lopes, 2009). Furthermore, KCNQ1 channel mutations associated with cardiac arrythmias in patients have been shown to affect PIP_2_-dependent activation, suggesting that native I_Ks_ channels are sensitive to PIP_2_ binding (Park et al., 2005; Li et al., 2011; Zaydman et al., 2013). A detailed understanding of the biophysical and molecular mechanisms by which PIP_2_ potentiates the function of KCNQ channels is an important step to understanding the physiology and pathophysiology of the cardiac, nervous, and auditory systems, and to developing effective new therapeutics for their diseases. In the following pages, we will review and attempt to synthesize the current body of work studying these mechanisms. Although the gating properties of KCNQ channels can be very different and accessory subunits lead to further variation, we will consider evidence regarding different KCNQ family members as well as KCNQ1 channels associated with KCNE1 accessory subunits. As described below, all these channels are voltage-gated, they all require PIP_2_ to open, and it appears that they all share a conserved PIP_2_ binding site at the VSD-PGD interface (see below). Therefore, we believe that considering these studies together may provide additional insights and may lead the reader to consider shared fundamental mechanisms that can tested and validated experimentally.

## Voltage-gated ion channel structure and function

**Figure 1:**
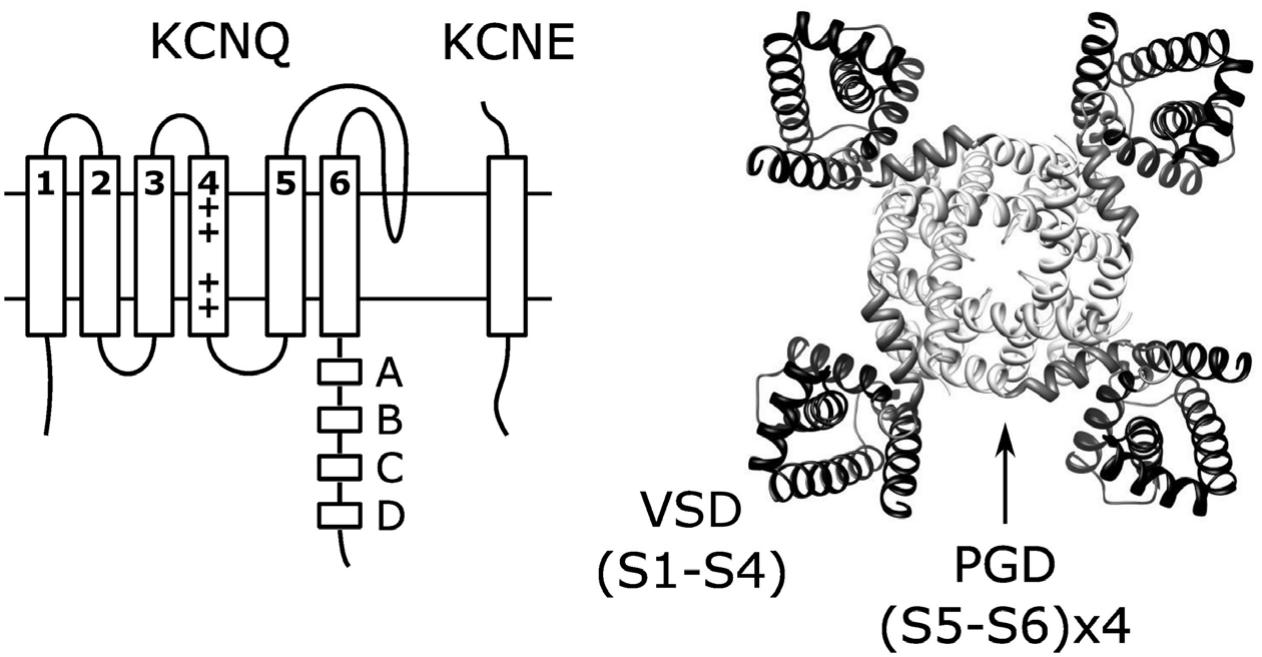
**Topology of single KCNQ subunit and the structure (right) of KCNQ channels formed by coassembly of four KCNQ subunits**. The structure shown is the KCNQ1 homology model (Smith et al., 2007) based on the Kv1.2 crystal structure (Long et al., 2005).

Voltage-gated cation channels share a common core structure consisting of four voltage-sensing domains (VSDs) surrounding a central pore-gate domain (PGD). In voltage-gated potassium (Kv) channels, including the members of the KCNQ (Kv7) family, tetrameric assembly of Kv-α subunits yields this channel structure (Figure 1). Each Kv-α subunit contains six transmembrane segments, of which S1-S4 from each Kv-α subunit form a VSD, while the S5-S6s from all four Kv-α subunits form the PGD. In general terms, voltage-dependent gating involves three different processes: VSD activation, PGD opening, and VSD-PGD coupling. In VSD activation, a conformational change is directly driven by transmembrane voltage. The voltage-dependence of this transition arises from forces that the transmembrane electric field exerts on conserved basic residues in S4 (Bezanilla, 2008). Depolarization yields outward force and promotes displacement of S4 from the resting to the activated state. The PGD forms a gated pathway through the membrane for selected ions to flow down their electrochemical gradient. The PGD opens by a dilation of the pathway at the crossing of the C-terminal portions of the S6 segments (the S6 gate) (Yellen, 1998). Several lines of evidence show that the VSD and the PGD are modular units. First, crystal structures of voltage-dependent cation channels reveal a surprisingly limited protein-protein contact surface at the VSD-PGD interface (Long, 2005; Payandeh et al., 2011). Second, voltage-independent channels consisting of the PGD alone without a VSD are found in nature, and they likely share a common, PGD-only ancestor with voltage-gated channels (Nayak et al., 2009). Likewise, voltage-sensor only proteins (VSOP) containing a functioning VSD without a PGD have been identified (Murata et al., 2005; Ramsey et al., 2006; Sasaki, 2006). Third, channels consisting of an artificially isolated PGD from voltage-gated potassium (Santos et al., 2008, 2012) or sodium (Shaya et al., 2011) channels have been shown to fold and function. Likewise, the artificially isolated VSD from KvAP adopts a similar conformation as in various crystal structures of full-length Kv channels (Butterwick and MacKinnon, 2010). Finally, functional, voltage-gated ion channels have been engineered by fusing together VSDs and PGDs from different sources (Lu et al., 2001; Arrigoni et al., 2013).

In VSD-PGD coupling, the conformations of the two modules are linked so that VSD activation promotes PGD opening, yielding a voltage-dependent conductance. Previous studies have identified several sites at the VSD-PGD interface that are important for the coupling, including the interactions between the S4-S5 linker and S6 (Lu et al., 2001, 2002; Tristani-Firouzi et al., 2002; Long, 2005; Haddad and Blunck, 2011), S4 and S5 (Ledwell and Aldrich, 1999; Soler-Llavina et al., 2006; Grabe et al., 2007), and S1 and the outer vestibule (Lee et al., 2009). Relative to what is known about VSD activation and PGD opening, their coupling remains poorly understood and the literature is largely focused on the protein-protein interactions. As will described below, PIP_2_ was recently found as an absolutely required cofactor for coupling between the activated state of the VSD and the open state of the PGD in KCNQ1 channels (Zaydman et al., 2013), providing a new opportunity for studying this critical, yet mysterious, process. Interestingly, the Kv1.2-2.1 crystal structure contains several anionic lipids bound stably at the VSD-PGD interface (Long et al., 2007), suggesting that lipid involvement in VSD-PGD coupling may be a principle common to Kv channels.

## Models of voltage-dependent gating and the description of VSD-PGD coupling

**Figure 2:**
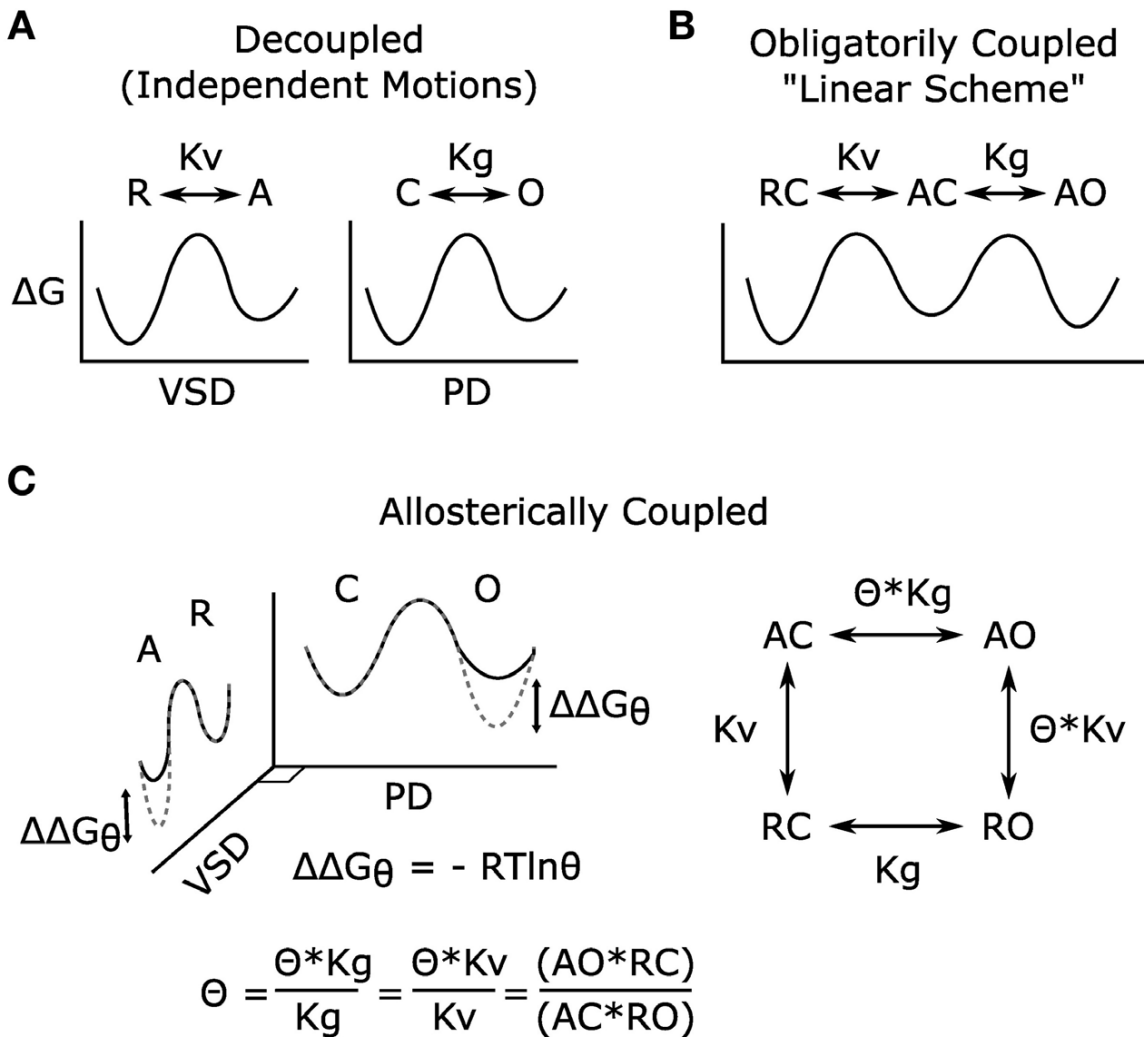
**Models of voltage-dependent gating**. **(A)** The VSD and PGD are independent structural and functional domains; each domain on its own may undergo conformational transitions that can be described by independent energy landscapes. These hypothetical reaction coordinates illustrate the free energy associated with the different states of the domains and the transitions among them. In the voltage-gated ion channel, the conformational changes of these domains are coupled, and such coupling has been modeled in two different ways **(B,C)**. Here, for simplicity, all models are shown considering only one VSD, and both VSD activation and PGD opening are modeled as two-state processes, for full models see references (Zagotta et al., 1994; Horrigan and Aldrich, 1999). **(B)** Linear gating scheme in which VSD activation obligatorily precedes PGD opening, and coupling is not explicitly defined. **(C)** Allosteric gating scheme in which the distinctive nature of the two domains is represented, and the coupling is explicitly defined. In this simple model, the coupling factor (θ) has been normalized to represent the net coupling that is due to interactions among all states of the two domains and does not represent a single physical interaction (Chowdhury and Chanda, 2012). The effects of this coupling can be represented in the energy landscapes **(C)** where the difference between the dotted-gray and solid-black lines represent the free energy changes due to the VSD-PGD coupling. For example, within the PGD plane, solid-black and dotted-gray indicate the free energy landscape for PGD opening when the VSD is resting or activated, respectively. R, resting VSD; A, activated VSD; Kv, equilibrium constant for VSD activation; C, closed PGD; O, open PGD; Kg, equilibrium constant for PGD opening; Θ, allosteric coupling factor; ΔG, free energy of gating; ΔΔG_θ_, free energy of coupling. As illustrated **(C)**, θ describes how much the open-close equilibrium is biased toward open when the VSD is activated. Equivalently, coupling can be quantified by measuring the effect of PGD opening on VSD activation, a measurement that is often more experimentally tractable (Arcisio-Miranda et al., 2010; Ryu and Yellen, 2012; Zaydman et al., 2013).

Given that distinct VSD and PGD domains form Kv channels and that coupling is essential to voltage-dependent gating (Figure 2A), any model describing voltage-dependent gating should explicitly define coupling. Two mathematical models of voltage-dependent gating have found widespread application. A linear activation scheme (see Figure 2B for a simplified version) was first used to describe the gating of Shaker potassium channels (Schoppa et al., 1992; Zagotta et al., 1994). In this model, PGD opening occurs only after the independent activation of the four VSDs. In contrast, an allosteric scheme (see Figure 2C for a simplified version) was devised to describe the voltage-dependent gating (in the absence of calcium) of calcium-activated, large conductance potassium (BK) channels (Horrigan et al., 1999). In the allosteric model, the PGD can open or close when the VSDs are either activated or resting; however, the coupling interactions between the two domains make PGD opening relatively more likely when the VSDs are activated. The linear model is compact and intuitive, and fits data well in special cases where PGD opening when the VSDs are resting is extremely low, as shown in wt Shaker channels (Islas and Sigworth, 1999). In fact, under these specific conditions the allosteric model is well approximated by the linear scheme (for an excellent review of this point, see Chowdhury and Chanda, 2012). However, the linear scheme has often been applied to voltage-gated channels for which these special condition have not been verified, which can lead to possible errors in the interpretation and analysis of data. Given the evidence for the independent structures and functions of the VSD and PGD, the allosteric model better reflects the physical reality by allowing for resting-open (RO, Figure 2) and activated-closed (AC) conformations. Although it appears more complex, the allosteric model provides the conceptual framework required by allowing a mathematical definition of coupling (θ in Figure 2C). Even still, the scheme in Figure 2C is overly simple in that the θ parameter is a complex normalized term, it is influenced by the interactions between all states of the VSD and PGD. Furthermore, as a result of normalization, the gating parameters (Kv, Kg) are also a function of the domain-domain interactions and do not reflect the intrinsic gating of the two domains in isolation (See Chowdhury and Chanda, 2012 JGP for more details).

Several lines of experimental evidence strongly support allosteric coupling of KCNQ channels. First, even at low voltages, wt KCNQ1 channels display a small, but measurable, conductance (~5% of maximal conductance), which is sensitive to mutations of KCNQ1 and a KCNQ channel blocker, indicating that it is indeed a KCNQ1 conductance (Ma et al., 2011). Second, KCNQ1 channels display no separation between the voltage-dependence of VSD activation and conductance, indicating that KCNQ1 channel opening does not require prior activation of all four VSDs (Osteen et al., 2010). Studies in which WT and mutant KCNQ1 subunits are mixed provided additional evidence that channel opening does not require the prior activation of all four VSDs (Meisel et al., 2012; Osteen et al., 2012). Third, locking the KCNQ1 channel PGD in an open-state does not prevent the VSD from transitioning between resting and activated states (Zaydman et al., 2013), providing direct evidence against the obligatory coupling imposed by linear activation schemes (Figure 2B) These studies revealed allosteric coupling in KCNQ1. M-channels formed by KCNQ2 and KCNQ3 subunits may also have allosteric coupling as suggested by the report that these channels can open even when only the high affinity KCNQ3 subunits are bound to PIP_2_ (Telezhkin et al., 2012a), suggesting that opening is not concerted as in the linear scheme (Figure 2B). Interpretation of experimental data through the lenses of the linear vs. allosteric gating models can resolve some of the differences between the biophysical and molecular models that have been proposed for the regulation of KCNQ channels by PIP_2_, as described below.

## Regulation of voltage-dependent gating by cofactor lipids

How might specific lipid interactions affect voltage-dependent gating? Three possible mechanisms are suggested above: by affecting VSD activation, PGD opening, and/or VSD-PGD coupling. Foundational work establishing roles for lipids as cofactors in voltage-dependent gating came through the study of Kv channel interactions with phospholipids other than PIP_2_. These findings are presented here, briefly.

MacKinnon and colleagues showed that, to open in response to depolarization, KvAP channels reconstituted into a planar bilayer require phospholipids in the membrane. They further found that, in sustaining current, the lipid phosphate group is key, not the net headgroup charge, the acyl chain length, or the effects of the lipids on membrane curvature (Schmidt et al., 2006). These findings suggest that specific interactions between the lipid phosphodiesters and the channel protein are important for voltage-dependent gating. Along these lines, crystal structures and molecular dynamics simulations of Kv channels reveal probable interactions between the S4 arginines and the lipid phosphate groups, which may help to stabilize the activated-state of the VSD (Freites, 2005; Long, 2005; Treptow and Tarek, 2006; Delemotte et al., 2011; Jensen et al., 2012). Zheng et al. used an antibody to the S3b-S4 paddle motif to detect VSD activation in reconstituted KvAP channels. They found that phospholipids are required for the antibody to bind at 0 mV, indicating that phospholipids are required for VSD activation to occur at this voltage. However, these effects were not dependent on the charge at the first two-conserved arginine positions, arguing against a role for specific interaction between these gating charges and the lipid phosphodiesters (Zheng et al., 2011). While the structural and molecular dynamics studies support the possibility of a direct effect on VSD activation, the functional data do not rule out alternative mechanisms, such as indirect effects on VSD activation through direct modulation of PGD opening or VSD-PGD coupling, or through non-specific lipid effects.

Another line of evidence for lipid modification of voltage-dependent gating comes from enzymatic modification of the headgroup of sphingomyelin, a zwitterionic lipid found primarily in the external leaflet of the cell membrane. Shingomyelinase D (Smase D) cleaves choline from the headgroup, leaving behind negatively charged ceramide-1-phosphate. Smase D treatment of oocytes expressing Kv channels was shown to left-shift the voltage dependence of PGD opening and VSD activation (Ramu et al., 2006). Again, these results alone do not provide exclusive evidence for a direct effect on VSD activation, as the voltage-dependence of VSD movement could be indirectly affected by alteration in the coupling or PGD opening (Colquhoun, 1998). However, Smase D was subsequently shown to left shift VSD activation in the voltage-sensitive enzyme CiVSP (Milescu et al., 2009). Provided that sphingomyelin acts by directly binding the channel, and not through aggregation of sphingomyelin-dependent lipid microdomains (Lingwood and Simons, 2009), this result provides direct evidence for an interaction between the sphongomyelin headgroup and the VSD, given that CiVSP contains only a VSD in its transmembrane domain (Murata et al., 2005). Sphingomyelinase C (Smase C), an enzyme that cleaves sphingomyelin after the phosphate, leaving behind a neutral headgroup, robustly inhibits the ionic current and gating charge movement in Kv channels. Unexpectedly, Smase C also inhibits currents from voltage-independent K^+^ channels (Xu et al., 2008) containing only a PGD in their transmembrane domain. This result suggests that sphingomyelin can act by directly affecting PGD opening, or through a non-specific mechanism.

Additional evidence for lipid interaction with the PGD comes from the presence of a lipid, phosphatidylglycerol (PG), in the crystal structure of the voltage-independent KCSA channel (Valiyaveetil et al., 2002), which is required for channel opening (Heginbotham, 1998; Valiyaveetil et al., 2002).

These studies have greatly enriched our understanding of the lipid regulation of voltage-dependent gating, but many questions remain unresolved. Particularly the role of lipids in VSD-PGD coupling was not explored. Previously, this coupling has been largely attributed to protein-protein interactions at the VSD-PGD interface. However, the presence of an anionic phospholipid bound at this interface in the Kv1.2/2.1 chimera channel crystal structure suggested that lipids may play a role as well (Long et al., 2007). Rodriguez-Menchaca et al. showed that mutations of the Kv1.2 S4-S5 linker, a structure that has been implicated in VSD-PGD coupling, increase the magnitude of GV shift and current inhibition caused by PIP_2_ depletion (Rodriguez-Menchaca et al., 2012). However, the effects of PIP_2_ on such Shaker-type channels are slight and complex (Abderemane-Ali et al., 2012; Rodriguez-Menchaca et al., 2012), and no biophysical mechanism has been established to explain these data. In contrast, PIP_2_ depletion completely inhibits the KCNQ currents, making KCNQ channels an excellent model system. We have recently developed an assay to directly detect the VSD-PGD coupling in KCNQ1 by using a mutation to lock the PGD in the open conformation and measuring the impact of PGD opening on VSD activation using voltage clamp fluorometry (VCF) (Zaydman et al., 2013). We found that the coupling between the activated-state of the VSD and the open-state of the PGD requires binding of PIP_2_ at the VSD-PGD interface (Figure 4), thereby providing functional evidence that lipids play a role in VSD-PGD coupling. The resemblance of the putative PIP_2_ binding site at the VSD-PGD interface of KCNQ1 to the anionic lipid-binding site in the Kv1.2/2.1 chimera channel structure (Long et al., 2007) suggests that lipid-mediated coupling may be a general property of voltage-gated channels. In the following pages we discuss these findings in the context of the large body of work on KCNQ-PIP_2_ interactions, generated by the efforts of many laboratories over the past decade.

## PIP_2_ is a KCNQ gating modulator

All five members of the KCNQ family require PIP_2_ to conduct measurable current (Zhang et al., 2003). KCNQ currents are inhibited by various treatments that decrease the abundance of PIP_2_ in the membrane, including stimulating PIP_2_ hydrolysis through receptor-mediated activation of phospholipase C (Suh and Hille, 2002; Zhang et al., 2003), elevating the rate of PIP_2_ dephopsphorylation through activation of lipid phosphatases (Suh et al., 2006; Murata and Okamura, 2007; Li et al., 2011; Kruse et al., 2012; Zaydman et al., 2013), suppressing PIP_2_ generation by inhibiting PIP kinases (Zhang et al., 2003; Shen, 2005), or directly chelating free PIP_2_ by applying PIP_2_ antibodies or polycations (ex. Mg^2+^, polylysine) (Suh and Hille, 2007b; Piron et al., 2010). Additionally, KCNQ currents undergo a spontaneous PIP_2_-dependent rundown in excised patches, which can be prevented or reversed through application of exogenous PIP_2_ (Loussouarn et al., 2003; Zhang et al., 2003; Li et al., 2005, 2011; Telezhkin et al., 2012a,b, 2013; Zaydman et al., 2013). These results show that PIP_2_ is required to sustain KCNQ channel currents. As the magnitude of the macroscopic current is a function of multiple parameters, these results could indicate that PIP_2_ affects the number of channels in the membrane, the single channel conductance, the probability of opening (gating), or the driving force for selected ions (Zaydman et al., 2012). The fast kinetics of current inhibition upon PIP_2_ depletion (Zhang et al., 2003; Falkenburger et al., 2010) and the reversibility of PIP_2_ activation of KCNQ channels in excised patches (Loussouarn et al., 2003; Zhang et al., 2003; Li et al., 2005, 2011; Telezhkin et al., 2012a,b, 2013; Zaydman et al., 2013) suggest a mechanism that is independent of the number of channels in the membrane. In single channel recordings of KCNQ2, KCNQ3, KCNQ4, and KCNQ2/KCNQ3 channels, the open probability approaches zero when PIP_2_ is depleted, and it increases, in a dose-dependent manner, to approach unity at PIP_2_ saturation. On the other hand, the slope conductance was found to be insensitive to PIP_2_, indicating that PIP_2_ does not change the single channel conductance or ionic selectivity (Li et al., 2005). These experiments show that PIP_2_ is required for gating of KCNQ channels.

KCNQ channels are generally known to be voltage-gated, but the effects of PIP_2_ on their voltage-dependency may appear different among KCNQ channels. Following muscarinic stimulation to partially deplete PIP_2_, Shapiro et al. found that conductance-voltage (GV) relationship of KCNQ2+KCNQ3 channels was unaffected (Shapiro et al., 2000). Subsequently, Nakajo and Kubo observed a rightward shift in the GV curve following activation of muscarinic receptors for KCNQ2, KCNQ2+KCNQ3 or KCNQ4 channels. However, this shift was fully accounted for by the downstream activation of PKC, and application of Wortmannin, a relatively unspecific kinase inhibitor that is known to block the PI 4- kinase (Nakanishi et al., 1995), did not affect the GV curves of homomeric KCNQ1 or KCNQ2 channels (Nakajo and Kubo, 2005). Finally, Hille and colleagues reported that increasing membrane PIP_2_ using inducible kinases did not shift the GV relationship for KCNQ2+KCNQ3 channels (Suh et al., 2006). On the other hand, when Loussouarn et al. excised membrane patches from COS-7 cells expressing KCNQ1 subunits with KCNE1 accessory subunits into bath solution containing high concentrations of PIP_2_ (5 μ g/mL), they observed a small (ΔV_1/2_ ~12 mV) leftward shift in the GV relationship and a slowing of the deactivation kinetics (Loussouarn et al., 2003). They also observed a hastening of the deactivation kinetics during spontaneous rundown in PIP_2_ free solutions (Loussouarn et al., 2003). Similarly, Li et al. (2011) excised membrane patches from *Xenopus oocytes* expressing KCNQ1 and KCNE1 subunits into bath solutions containing high (300 μ M) PIP_2_ and observed a left shift in the GV (ΔV_1/2_ ~13 mV); however, no changes of deactivation kinetics were observed under these conditions or during spontaneous PIP_2_-dependent rundown. These seemingly contradictory kinetic results can be explained by different levels of expression of the KCNE1 subunit in these two studies. The currents recorded by Loussouarn et al. opened at hyperpolarized potentials (V_1/2_on cell ~−34 mV) and displayed a prominent tail hook indicative of inactivation. These are two hallmarks of homomeric KCNQ1 channels that are not usually present when KCNE1 is coexpressed (Barhanin et al., 1996; Sanguinetti et al., 1996; Pusch et al., 1998). As Loussouarn et al. measured deactivation at −40 mV, which is just below the half maximal activation, the effect of PIP_2_ on deactivation kinetics may be attributed to the shift in steady-state activation. Specifically, the deactivation slows at high PIP_2_ because −40 mV point moves higher up the steady-state activation curve, and vice versa. In contrast, the currents recorded by Li et al. manifested at more depolarized voltages (V_1/2_ on cell = +21.6 ± 3.2 mV) and did not display a tail hook, consistent with robust expression of KCNE1. Furthermore, Li et al. measured deactivation kinetics at −80 mV, at which point steady-state opening is minimal under conditions of both low and high PIP_2_. This result suggests, when isolated from activation, deactivation kinetics are insensitive to PIP_2_. While PIP_2_-dependent GV shifts are mild for wt KCNQ1 + wt KCNE1, the shift is greater (ΔV_1/2_ = −40 mV: on cell vs. 300 μM PIP_2_) when wt KCNQ1 is expressed with a mutant KCNE1 - K70N (Li et al., 2011). Several mutations of KCNQ1 or KCNE1, including K70N, right-shift the GV relationship of KCNQ1+KCNE1 channels, and they decrease the apparent affinity of KCNQ1+KCNE1 channels for exogenous PIP_2_ (Park et al., 2005; Li et al., 2011) or decrease the binding of C-terminal fragments of KCNQ1 to immobilized PIP_2_ (Thomas et al., 2011). Of these mutations, it has been shown for K70N that application of supranormal levels (300 μ M) of PIP_2_ reverses the shift, so that the GV relationship can be superimposed on that of wt channels under identical conditions (Li et al., 2011). These data suggest that the effects of K70N on PIP_2_ sensitivity can fully account for the shift in voltage-dependence. Altogether, these results provide evidence that PIP_2_ affects the voltage-dependent gating of KCNQ1+KCNE1 channels. It is important to bear in mind that the lack of a GV shift for other KCNQ channels, including homomeric KCNQ1, does not rule out an effect of PIP_2_ on their voltage-dependent gating (see below).

## Biophysical models for PIP_2_ regulation of voltage-dependent gating in KCNQ

As described above, voltage-dependent gating involves three fundamental processes: VSD activation, VSD-PGD coupling, and PGD opening. Recently, we have investigated which step(s) requires PIP_2_ in KCNQ1 channels. To test if PIP_2_ affects VSD activation in KCNQ1 channels, VSD activation was detected by using voltage clamp fluorometry (VCF) (Mannuzzu et al., 1996; Osteen et al., 2010) while dynamically depleting the endogenous PIP_2_ by expressing and activating the lipid-phosphatase CiVSP (Murata et al., 2005; Murata and Okamura, 2007). PIP_2_ depletion did not prevent movement of the VSD in response to membrane voltage; meanwhile, the ionic currents were robustly inhibited (Zaydman et al., 2013). Furthermore, the fluorescence-voltage (FV) relationship, a measure of the steady-state voltage-dependence of VSD activation, was insensitive to PIP_2_ depletion suggesting that VSD movements are not directly affected by PIP_2_. As a complementary method to VCF, we detected VSD activation as a voltage-dependent conformational change altering the chemical accessibility of the residue I230C within S4 to extracellular MTSES. Modification of I230C is voltage-dependent in the presence of endogenous PIP_2_ and after PIP_2_ depletion by CiVSP, confirming that PIP_2_ is not required for VSD activation in KCNQ1 (Zaydman et al., 2013). These results suggest that PIP_2_ is required for either PGD opening or VSD-PGD coupling.

We then tested if PIP_2_ is required for coupling by measuring the impact of PGD opening on VSD activation both in the presence of endogenous PIP_2_ and after PIP_2_ depletion by CiVSP. In the presence of PIP_2_, VSD-PGD coupling caused a leftward shift in the FV relationship when the PGD was locked open by the mutation L353K. After depletion of PIP_2_, L353K KCNQ1 channels remained open, but the shift was eliminated and the FV relationships of L353K and wt could be superimposed (Zaydman et al., 2013). This result demonstrates that PIP_2_ is required for PGD opening to promote VSD activation, in addition to being required for VSD activation to promote PGD opening. These bidirectional effects are consistent with a model in which PIP_2_ is required for allosteric coupling between the activated-state of the VSD and the open-state of the PGD. A simple version of such a model globally fits the steady-state voltage- and PIP_2_-dependencies of PGD opening and VSD activation in KCNQ1 (Zaydman et al., 2013). This model demonstrates that the effect of PIP_2_ on coupling is sufficient to quantitatively model the gating behavior of KCNQ1 without any direct effects on PGD opening; however, these results do not rule out the possibility that PIP_2_ affects PGD opening as well.

Loussouarn et al. proposed a model for PIP_2_ regulation of KCNQ1+KCNE1 channels in which the role of PIP_2_ is to stabilize the PGD open-state (Loussouarn et al., 2003). This model recapitulated their observations that PIP_2_ left-shifts the GV relationship and slows current deactivation without changing the kinetics of current activation. This model is in agreement with the model of Zaydman et al. in the sense that PIP_2_ does not directly affect VSD activation; however, the linear architecture of this model does not allow one to identify effects on VSD-PGD coupling independently of PGD opening. Furthermore, it is debatable if deactivation kinetics are directly sensitive to PIP_2_ (see above). Loussouarn et al. draw an insightful analogy to voltage-independent Kir channels for which PIP_2_ binding at the C-terminus of the PGD has been suggested to stabilize the PGD open-state. However, recent crystallographic evidence suggests that PIP_2_ mediates coupling between the pore and cytosolic domains, not PGD opening, in Kir channels (Hansen et al., 2011; Whorton and MacKinnon, 2011). To the best of our knowledge, there is no firm evidence for or against a direct effect of PIP_2_ binding on the PGD opening in a KCNQ channel.

## Molecular models of KCNQ-PIP_2_ interaction

**Figure 3:**
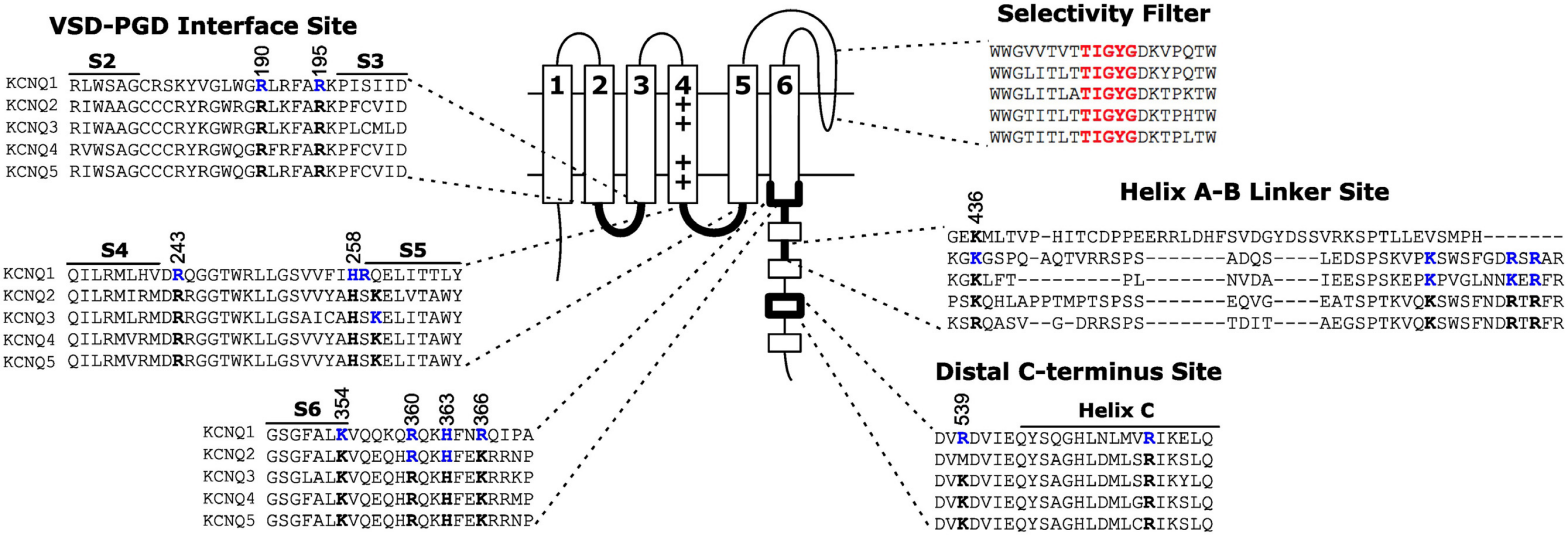
**Location of three proposed PIP_2_ interacting sites on KCNQ**. The VSD-PGD interface site includes contributions from the S2-S3 linker, S4-S5 linker, S6 and proximal C-terminus. Other sites have been proposed at the helix A-B linker or the distal C-terminus. Blue residues indicate residues for which mutations have been reported to affect PIP_2_ dependent activation. Black residues highlight the conservation of such residues among other members of the KCNQ family. Numbering indicates the positions of highlighted residues in the human KCNQ1 channel sequence.

Identification of the binding site is critical to mechanistically understanding how PIP_2_ regulates KCNQ channels, and may hold the key to developing new therapeutics targeting PIP_2_-dependent activation. The search for putative PIP_2_ interacting partners has focused on basic residues that are exposed to the cytosol based on the rationale that PIP_2_ is restricted to the intracellular leaflet of the membrane and that crystalographically verified PIP_2_ binding sites in other proteins contain multiple basic residues for coordination of the negatively charged headgroup phosphates (McLaughlin et al., 2002; Rosenhouse-Dantsker and Logothetis, 2007; Suh and Hille, 2008; Hansen et al., 2011; Whorton and MacKinnon, 2011). The importance of electrostatic interactions on PIP_2_ activation of KCNQ has been verified by Brown and colleagues by studying the ability of various exogenous lipids to sustain the single channel open probability of KCNQ2+KCNQ3 channels at 0 mV. They found that a phosphate on the lipid headgroup is required, and the apparent affinity to different phosphoinositide species increases with headgroup charge; i.e., more phosphates yield higher apparent affinity (Telezhkin et al., 2012b). On the other hand, synthesized dic4-PIP_2_, dic8-PIP_2_, and long-chain PIP_2_ purified from bovine brain have similar effects on the activation of KCNQ1 channels (Li et al., 2011), suggesting that the acyl chains of PIP_2_ molecules do not affect the extent of channel activation. Implicit in the logic of studying basic residues is the idea that they are participating in a direct electrostatic interaction with the PIP_2_ headgroup. However, a high level of caution is required when putative binding residues are identified through functional studies because both binding and gating will determine the apparent affinity (for an excellent review of this topic, see Colquhoun, 1998). Many residues have been proposed to be important for the PIP_2_ dependent activation of KCNQ channels, which can be grouped into three sites: the VSD-PGD interface site, the helix A-B linker site, and the distal C-terminus site (Figure 3).

**Figure 4:**
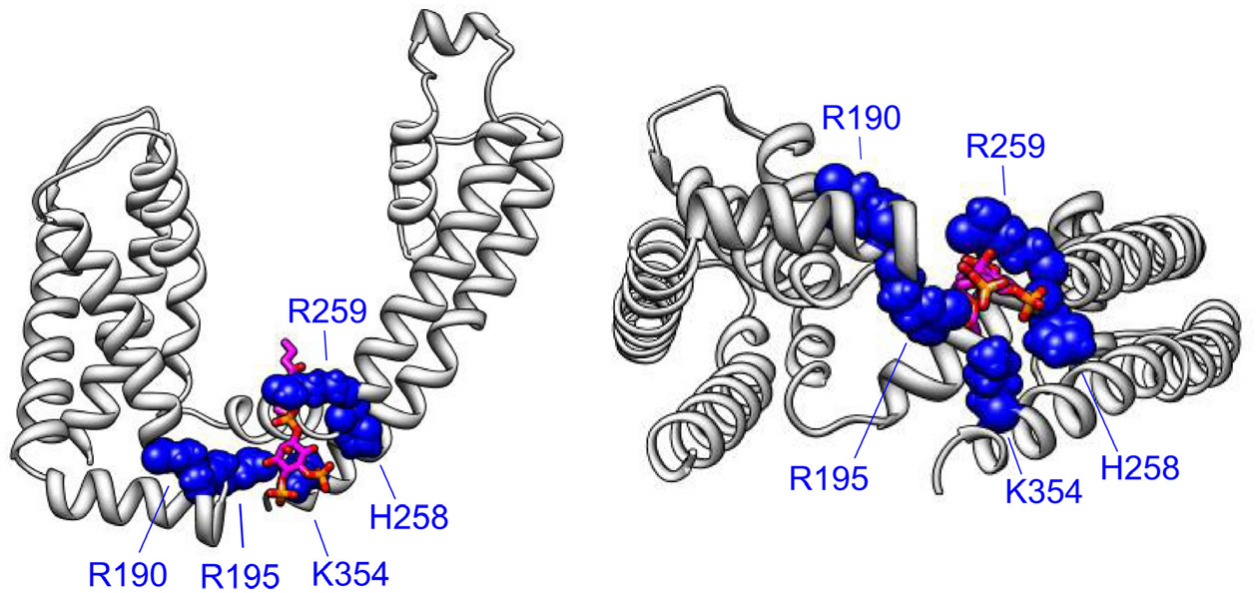
**The VSD-PGD interface site**. Side view (left) and bottom view (right) of a single KCNQ1 subunit from a homology model (Smith et al., 2007), which was built of the template of the Kv1.2 crystal structure (Long et al., 2005). PIP_2_ molecule (magenta, orange, and red) is positioned by aligning the Kir2.2-PIP_2_ crystal structure (Hansen et al., 2011) as done previously (Zaydman et al., 2013). Important residues for PIP_2_-dependent coupling are shown. Note that some additional residues reside in more C-terminal regions that are not represented in the homology model due to a lack of a structural template for the KCNQ C-terminus.

The VSD-PGD interface site includes contributions from the S2-S3 linker, S4-S5 linker and the proximal C-terminus (Figure 3). Logothetis and colleagues identified a mutation, H328C, in the proximal C-terminus of KCNQ2, that significantly decreased the apparent affinity of KCNQ2+KCNQ3 channels to exogenous PIP_2_ (Zhang et al., 2003). Subsequently, Loussouarn and colleagues demonstrated that the disease-associated mutation R243 in the S4-S5 linker decreases the apparent affinity of KCNQ1+KCNE1 channels for exogenous PIP_2_ (Park et al., 2005). Recently, Li and colleagues reported that K222A, in the S4-S5 linker, lowered the apparent PIP_2_ affinity of KCNQ3 channels (Zhou et al., 2013). In 2011, Tinker and colleagues found a cluster of basic residues (K354, K358, R360, K362) in the KCNQ1 proximal C-terminus is important for the binding of C-terminal fragments of KCNQ1 to immobilized phosphoinositides (Thomas et al., 2011). One concern with such an approach is that binding of a protein fragment may represent a non-specific electrostatic interaction that is not representative of PIP_2_ binding in the channel protein. Accordingly, these authors found that the identified mutations decreased the whole cell currents of full-length KCNQ1+KCNE1 channels, and a double mutation (K358A/R360A) had a blunted response to dialysis of 25 μ M diC8 PIP_2_. Studying the homologous residues in KCNQ2, Brown and colleagues found that the mutation R325A decreased the on-cell, single-channel open probability and the apparent affinity for diC8 PIP_2_ in excised patches; in contrast, the mutation K327A did not affect the dose response of KCNQ2 (Telezhkin et al., 2013). Lopes and colleagues found that the long QT syndrome-associated mutation R366Q increased the sensitivity of KCNQ1+KCNE1 channels to PIP_2_ hydrolysis (Matavel et al., 2010). We have recently shown that charge neutralizing and charge reversing mutations of basic residues at the VSD-PGD interface affect the whole cell current amplitude in a manner that is correlated with their effects on apparent PIP_2_ affinity and PIP_2_-dependent VSD-PGD coupling. Of particular interest, mutations of eight residues in KCNQ1 essentially eliminate VSD-PGD coupling and the whole cell current without preventing channel expression in the membrane or VSD activation (Zaydman et al., 2013). These residues are located in the S2-S3 linker (R190, R195), the S4-S5 linker (H258, R259) and the proximal C-terminus (K354, R360, H363, and R366). The residues at the end of S6 overlapped with some proposed earlier by other groups (Zhang et al., 2003; Matavel et al., 2010; Thomas et al., 2011; Telezhkin et al., 2013). Alignment of a KCNQ1 homology model (Smith et al., 2007) with the Kir-PIP_2_ structures (Hansen et al., 2011; Whorton and MacKinnon, 2011) revealed remarkable overlap of the VSD-PGD interface site in KCNQ1 the Kir PIP_2_ binding site (Zaydman et al., 2012). This evidence suggests that the VSD-PGD interface site constitutes a true PIP_2_ binding site that bridges basic residues in the VSD and the proximal C-terminus (including the S6 gate) (Figure 4). This molecular model is consistent with the biophysical model that PIP_2_ is required for coupling in KCNQ1. Furthermore, the basic residues within the VSD-PGD interface site are well conserved among all KCNQ channels (Figure 3) suggesting that PIP_2_ may be required for VSD-PGD coupling in KCNQ2-KCNQ5 as well. Along these lines, in a recent molecular dynamics simulation of KCNQ2, Yang and colleagues observed state-dependent interactions between PIP_2_ and K162 in the S2-S3 linker and K230 in the S4-S5 linker, and they observed, in experiments, that mutations of these residues cause a change in the apparent PIP_2_ affinity for PIP_2_ (Zhang et al., 2013).

Shapiro and colleagues identified a possible interaction between the helix A-B linker of the C-terminus and PIP_2_ (Hernandez et al., 2008). They used a chimera approach to investigate the structural basis for the large difference in apparent PIP_2_ affinity between KCNQ3 and KCNQ4 channels. They found that chimeras that exchange portions of the C-terminus including the helix A-B linker yield chimeric channels with an apparent affinity that resembles the channel from which the A-B linker originates more than the background channel. For example, the KCNQ4 A-B linker confers wt KCNQ4-like sensitivity in the background of KCNQ3. The A-B linker contains several basic residues that are highly conserved among KCNQ2-KCNQ5 (Figure 3). Neutralizing and charge reversing mutations of these residues exhibit decreased on cell open probability, and a triple charge reversing mutation (K352E/R459E/R461E KCNQ2) decreases the apparent affinity to exogenous PIP_2_ (Hernandez et al., 2008) and the binding of KCNQ2 C-terminal fragments to immobilized phosphoinositides (Telezhkin et al., 2013). However, the critical A-B linker residues are not conserved in KCNQ1 (Figure 3), and chimeras replacing the KCNQ2 and KCNQ3 C-termini with that of KCNQ1 generates functional KCNQ2CTQ1/KCNQ3CTQ1 channels (Li et al., 2013). Furthermore, deletion of the A-B linker yields functional, PIP_2_-dependent KCNQ2 channels that are no more severely inhibited by PIP_2_ depletion than WT KCNQ2 (Aivar et al., 2012). These results suggest that the site at the A-B linker may not be the primary binding site for PIP_2_. Indeed, Brown and colleagues studied dic8-PIP_2_ dependence of the wild type KCNQ2/KCNQ3 and the wild type KCNQ2 coexpressed with the mutant KCNQ3 containing charge reversal of key residues in the A-B linker site, KCNQ2/KCNQ3(EEE), and suggested that the mutations in KCNQ3(EEE) do not change PIP_2_ binding affinity directly but reduce channel activation by markedly decreasing channel opening (Telezhkin et al., 2012a).

The residues in the distal C-terminus site (R539 and R555 in KCNQ1) were found by the Loussouarn group (Park et al., 2005). They studied the long QT syndrome-associated mutations R539W and R555C, located within helix C of the C-terminus, and found that these mutations decrease the apparent affinity KCNQ1+KCNE1 channels to exogenous PIP_2_. The distal position of R539 and R555 in the protein sequence makes it difficult to locate these residues with respect to the membrane because the structure of the large KCNQ C-terminus is unknown. There are several possible explanations for how these residues affect PIP_2_ dependent activation. First they could form an independent PIP_2_ binding site that affects channel gating. Second, the structure of the C-terminus could position these residues close to the VSD-PGD interface site to form a single PIP_2_ binding site. Third, the distal C-terminus site could be allosterically linked to a distant PIP_2_ binding site instead of directly coordinating PIP_2_. Further studies are required to address these possibilities.

## Coupling as a conserved mechanism for lipid modulation of transmembrane proteins

**Figure 5:**
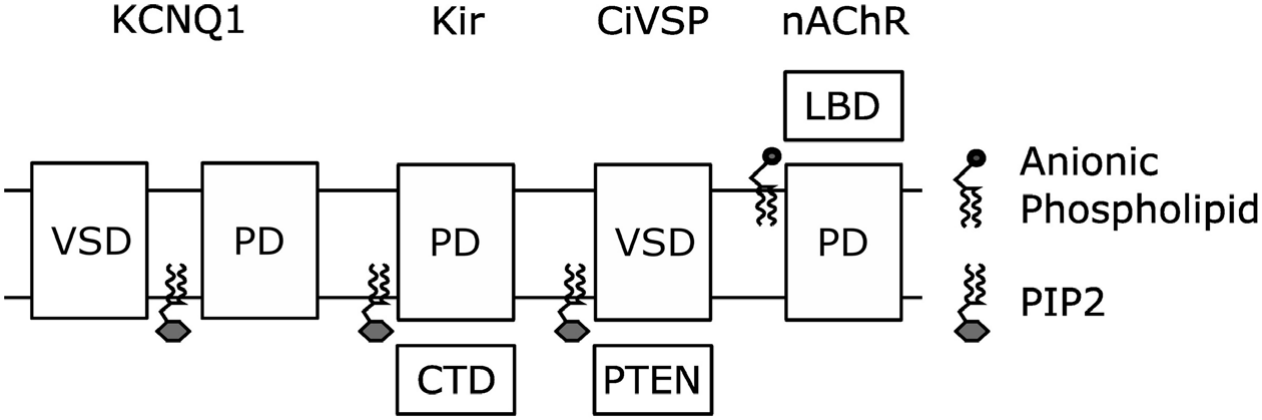
**Coupling of modular sensor and effector domains as a general mechanism for the regulation of transmembrane proteins by PIP_2_ or other anionic phospholipids**.

The remarkable similarity in the location of the putative PIP_2_ binding site in KCNQ1 and the PIP_2_ binding site in the Kir crystal structure (Zaydman et al., 2013) suggests a conserved mechanism for PIP_2_ regulation of voltage-dependent and voltage independent K^+^ channels. Consistently, recent structural studies suggest that PIP_2_ is required to couple the Kir cytosolic domain, a sensor of intracellular factors, to the PGD. The Kir2.2 structures reveal that PIP_2_ binding pulls the cystosolic domain toward the PGD by roughly six angstroms and causes them to engage in a set of interactions that moves the PGD toward an open conformation (Hansen et al., 2011). The Kir3.1 structures suggest that PIP_2_ binding in between the cytosolic domain and the PGD is required to couple opening of the intracellular gate within the cytosolic domain to opening of the PGD (Whorton and MacKinnon, 2011). Thus the activation of Kir channels by PIP_2_, which has been attributed to open-state stabilization using linear activation schemes, is likely due to potentiation of coupling between the cytosolic domain and the PGD. Therefore, coupling between modular sensor domains and the PGD could be a conserved mechanism for PIP_2_ regulation of ion channels. Along these lines, Kohout et al. showed that PIP_2_ is required for mutations of the active site of the CiVSP phosphatase domain to shift the voltage-dependence of VSD activation, indicating that PIP_2_-dependent coupling may extend beyond PIP_2_-sensitive ion channels to PIP_2_ sensitive transmembrane proteins (Kohout et al., 2010). Even further yet, De Costa et al. demonstrated that anionic lipids (not PIP_2_) are required to couple the conformation of the extracellular ligand-sensing domain of the nicotinic acetylcholine receptor to the conformation of the transmembrane pore (daCosta and Baenziger, 2009; daCosta et al., 2009). From all these studies emerges a common theme, cofactor lipids modulate the function of transmembrane proteins by potentiating the coupling between modular sensor and effector domains. In fact this collection of work demonstrates this point in nearly every combination: transmembrane sensor to cytosolic effector (CiVSP), cytosolic sensor to transmembrane effector (Kir), extracellular sensor to transmembrane effector (nAChr), and transmembrane sensor to transmembrane effector (KCNQ1) (Figure 5).

## PIP_2_ mediated coupling as a new therapeutic target

Voltage-gated ion channels are an appealing drug target (Kaczorowski et al., 2008). They are expressed in the surface membrane of many cells, directly determine cellular excitability, and couple to intracellular signaling pathways. Furthermore, a diverse set of diseases arise from inherited or acquired disturbances in ion channel function, and PIP_2_ sensitive channels have been implicated in many of these channelopathies (Logothetis et al., 2010). The predominant strategies for modulating voltage-gated ion channel function with small molecules have been targeted at VSD activation, PGD opening, or permeation, while VSD-PGD coupling has been relatively ignored. An argument can be made that VSD-PGD coupling is superior drug target. Theoretically, modulating coupling can increase or decrease the magnitude of the current without significantly changing the critical properties of voltage-dependence and time-dependence, as we have seen in the case of KCNQ1 (Zaydman et al., 2013). This is significant because voltage-gated ion channels often work in concert, and the voltage- and time- dependence of the current are critical to efficient and orderly excitation waveforms. With the discovery of PIP_2_ mediated VSD-PGD coupling in KCNQ1 and the characterization of the binding pocket for coupling (Zaydman et al., 2013) we now have a structural target for small molecules to modulate VSD-PGD coupling. Of course, many issues regarding safety, efficacy and specificity must be addressed before such a therapeutic can be brought to market. However, it is exciting that the compound zinc pyrithione has recently been shown to rescue KCNQ channel current following PIP_2_ depletion, compete with PIP_2_ for activation of KCNQ, and to lose activity when mutations are made to the putative PIP_2_ binding residues (Zhou et al., 2013). These results suggest that zinc pyrithione targets the VSD-PGD coupling that is typically mediated by PIP_2_ binding, in which case it would represent the first in its class.

### Conflict of interest statement

The authors declare that the research was conducted in the absence of any commercial or financial relationships that could be construed as a potential conflict of interest.
